# Outcomes of children treated for relapsed or refractory acute lymphoblastic leukemia: A single tertiary care center experience

**DOI:** 10.1002/cnr2.2117

**Published:** 2024-07-25

**Authors:** Mosfer AlMalki, Mohammed Abdulatef, Hassan Altrabolsi, Nasser Shubayr

**Affiliations:** ^1^ Oncology Department King Faisal Specialist Hospital and Research Centre Jeddah Saudi Arabia; ^2^ Department of Diagnostic Radiography Technology, College of Applied Medical Sciences Jazan University Jazan Saudi Arabia

**Keywords:** acute lymphoblastic leukemia, children, relapse, resistance, risk stratification, survival

## Abstract

**Background:**

Acute lymphoblastic leukemia (ALL) is one of the most common malignancies among children. Despite success in frontline treatment, 20% of children will relapse or show resistance to treatment.

**Aim:**

The aim of this study is to evaluate the clinical characteristics of children diagnosed and treated for refractory or relapsed ALL and determine 3‐year overall survival (OS) outcomes.

**Method:**

This study involved a retrospective chart review of patients aged 1–14 years diagnosed with ALL during January 2002 to December 2018. Data were extracted for baseline characteristics at diagnosis and at relapse.

**Results:**

A total of 347 newly diagnosed children with ALL were identified, among whom there were three induction failures (IF) and 28 relapses, resulting in a cohort of 31 patients with a relapse rate of 9%. The male‐to‐female ratio was 4.16:1, and the mean duration of first complete remission (CR1) was 26 months. Fifteen (48%) patients relapsed ≤18 months, 7 (23%) during 18–36 months, and 9 (29%) relapsed >36 months of IF or CR1. Nineteen patients (61%) had isolated bone marrow (BM) relapse, 7 (23%) patients experienced isolated extramedullary relapse (5 isolated CNS relapse and 2 isolated testicular relapse), and 5 (16%) patients experienced BM involvement with other sites (4 BM + CNS and 1 BM + testis). The 3‐year OS of the cohort was 62.3%, while in patients with CR post first‐salvage therapy, a 3‐year OS of 79.5% was observed (*p* value <.05 compared with patients who did not achieve remission post first‐salvage therapy, 3‐year OS: 46.4%). The same statistical difference was observed in 3‐year OS when comparing the duration of remission of CR prior to relapse: ≤18 months, 33.2%; 18–36 months, 66.7%; and >36 months, 87.5%. The same trend continued when comparing 3‐year OS based on risk stratification at relapse: low risk (LR), 83.3%; intermediate risk (IR), 80%; and high risk (HR), 44.8%.

**Conclusion:**

The incidence and outcomes reported are comparable to internationally reported data regarding the duration of CR1. Risk stratification at relapse and remission status post‐salvage therapy were identified as significant prognostic factors for survival. No survival difference was observed among patients who received hematopoietic stem cell transplantation after induction compared with those who received chemotherapy, which could be attributed to the smaller sample size, warranting a multi‐institutional observational study. These findings corroborate the need for novel therapies and treatment approaches.

## 1 INTRODUCTION

Acute lymphoblastic leukemia (ALL) stands as predominant malignancies in children, marked by the widespread invasion of bone marrow (BM) and peripheral blood by malignant cells. This disease accounts for more than a quarter of all pediatric cancer diagnoses.^1^, ^2^ The frontline treatment of ALL represents a significant triumph, positioning it among the most treatable cancers in the pediatric population, with an overall survival (OS) rate exceeding 80%. The achievement of favorable survival outcomes is credited to a multifaceted approach that encompasses a combination of chemotherapeutic agents, the implementation of risk‐based stratification for treatment allocation, and the administration of prophylactic therapy to the central nervous system (CNS).^3^, ^4^ Despite substantial advancements in treatment methodologies, a concerning estimate suggests that around 20% of patients may experience a relapse.^1^, ^3^

Identifying risk factors for relapse has become a pivotal aspect of pediatric oncology, aiding oncologists in devising treatment plans and prognostic assessments. These factors include the duration until relapse from the first complete remission (CR1), specifically if it occurs within 36 months of diagnosis, the relapse site, and the immunophenotype (B‐cell vs. T‐cell). The prognosis for relapsed patients is significantly influenced by the relapse site and the length of CR1. BM relapses and early recurrences are particularly associated with a less favorable prognosis compared with isolated extramedullary or late relapses. While achieving clinical remission (CR) in the majority of relapse cases is feasible, the long‐term survival rates fluctuate between 40% and 50%.^5^, ^6^

The treatment regimen for relapsed patients typically involves re‐induction with the same conventional agents used at initial diagnosis. Hematopoietic stem cell transplantation (HSCT) is frequently utilized as a consolidation therapy in these instances. Nonetheless, the efficacy of HSCT in cases of late or multiple relapses remains to be conclusively established. The suboptimal outcomes observed with conventional chemotherapy in the management of relapsed or refractory ALL underscore the urgent need for novel therapeutic agents and strategies.^3^, ^7^

This study focuses on the clinical characteristics of children diagnosed with refractory or relapsed ALL, aims to determine the 3‐year OS rate in this group, and seeks to compare risk‐stratified groups to identify prognostic factors. A risk‐stratified approach is employed at our institution for treating pediatric patients who have relapsed or shown resistance to first‐line therapy. Through this investigation, we present the outcomes from a single tertiary care center.

## 2 METHODS AND MATERIALS

### 2.1 Study design and population

This study is a retrospective analysis focusing on children aged 1–14 years diagnosed with ALL who experienced relapse after first‐line therapy or showed resistance to it. In this study, the term “refractory” or “resistant” to first‐line therapy was defined using clinical and hematological criteria. Patients were classified as refractory if, after initial treatment, they failed to achieve CR as evidenced by less than 5% blasts in the BM, presence of minimal residual disease (MRD) detected by flow cytometry or PCR indicating molecular‐level resistance, or experienced early relapse within 12 months post‐CR. The inclusion criteria encompassed patients treated for relapse at King Faisal Specialist Hospital & Research Centre in Jeddah, Saudi Arabia, from January 2002 to December 2018. The pediatric age group was defined as birth to 14 years, aligning with the hospital's practice. Exclusion criteria were patients with Down syndrome, a histopathological diagnosis of acute myeloid leukemia (AML) or mature B‐Cell ALL, secondary ALL, underlying BM failure syndromes, and those with incomplete medical records.

### 2.2 Ethical considerations

The study received approval from the institutional review board, adhering to the highest ethical standards. Data abstraction was conducted using an IRB‐approved case report form (CRF), ensuring the confidentiality and privacy of patient information. All procedures were in strict compliance with Good Clinical Practice (GCP) guidelines. Access to data was restricted to the research team through password‐protected accounts, safeguarding participant information throughout the study.

### 2.3 Data collection and extraction

The pediatric oncology research database was queried for patients diagnosed with ALL within the specified timeframe. Within the database, a total of 347 newly diagnosed children with ALL were initially identified according to the study‐defined inclusion and exclusion criteria. However, the focused study cohort was narrowed down to 31 children who experienced either relapse or resistance to first‐line therapy and subsequently underwent salvage therapy. The data of study cohort were extracted from medical records, focusing on baseline characteristics at diagnosis and at the point of relapse. The REDCap electronic data capture tool, hosted by the institution, was employed for data collection and management, ensuring a structured and secure process.

### 2.4 Criteria for CR and relapse

CR was defined by the presence of an M1 marrow (<5% blasts in bone marrow aspirate) following second‐line salvage induction therapy, absence of circulating blasts or extramedullary disease, and recovery of peripheral counts (ANC >0.75 × 10^9^/L and platelet count >75 × 10^9^/L). Failure to achieve CR post‐salvage therapy was classified as induction failure (IF). Subsequent relapses in patients previously in CR were pathologically confirmed by M3 marrow (≥25% leukemic blasts) or the presence of extramedullary disease. OS duration of the cohort for disease‐recurrence was determined from relapse date to date last seen and in case of a deceased patient date of demise was used, while OS of cohort for diagnosis was determined form diagnosis date to date last seen or date of demise in case of a deceased patient.

#### 2.4.1 First line therapy

Among the 31 treatment‐naïve patients receiving risk‐stratified first‐line therapy, three low‐risk (LR) patients with precursor B‐cell ALL, prior to 2008, were treated according to the CCG1891 protocol. This protocol comprises a three‐drug induction regimen (prednisone, vincristine, and asparaginase), along with CNS‐directed consolidation therapy and two delayed intensification phases. Ten patients identified as high‐risk (HR) were treated with the CCG‐1882 protocol, which includes a four‐drug induction regimen (prednisone, vincristine, asparaginase, and daunorubicin). The HR chemotherapy regimen featured more intensive consolidation and a single delayed phase of intensification. Intensified intrathecal therapy was provided to patients who did not receive cranial radiation therapy. Prophylactic cranial radiation therapy was administered to older children (aged over 10 years) without CNS disease, while craniospinal radiation was provided to those with CNS disease. Additionally, one patient with T‐cell ALL, categorized as HR before 2008, was treated with the St. Jude Total XIII B HR protocol.

From 2008 onwards, chemotherapy regimen modifications aligned with risk stratification, incorporating the RUNX1‐ETV6 translocation for the LR category and further treatment intensification. Five LR patients received the CCG1991 chemotherapy regimen. Additionally, one LR patient was treated with the COG‐AALL0232 protocol, which includes a four‐drug regimen (cytarabine, vincristine, daunorubicin, peg‐asparaginase, and prednisolone) with prolonged induction for those with M2 or M1 disease status and more than 1% MRD. Six HR patients received the CCG1961 regimen, and four HR patients were treated with the COG‐AALL0232 chemotherapy protocol. Furthermore, one HR patient with B‐cell ALL was treated with the St. Jude Total XIII B HR regimen. The post‐2008 treatment for HR patients also included three diagnosed with T‐cell ALL, emphasizing the tailored approach based on specific ALL subtypes and risk categories.

#### 2.4.2 Risk stratification at relapse

The study employed the risk‐stratification definitions established by the Children's Oncology Group (COG): LR encompasses cases of late B‐ALL marrow relapse and late isolated extra‐medullary (IEM) relapse, both with end‐block 1 MRD <0.1%; intermediate risk (IR) includes cases of late B‐ALL marrow and late IEM relapse, both with end‐block 1 MRD ≥0.1%; HR patients are characterized by early B‐ALL marrow relapse, early IEM relapse, and T‐Cell All relapse, regardless of timing and site. Late IEM relapse is defined as occurring ≥18 months from diagnosis, while early IEM relapse occurs <18 months from diagnosis. Early marrow relapse is defined as occurring <36 months from diagnosis, whereas late marrow relapse is defined as occurring ≥36 months from diagnosis.

#### 2.4.3 Second line therapy

The strategy for second‐line therapy was established based on a study‐specific risk assessment, identifying patients who demonstrated resistance to the initial four‐drug regimen. These patients were divided into two groups for re‐induction therapy. The first group, comprising six patients, received the CCG regimen‐A, which included high‐dose cytosine arabinoside (HD‐AraC) in combination with either fludarabine or idarubicin. The second group, also consisting of six patients who had a CR1 duration of 18 months, were reinduced with CCG regimen‐B that utilized a four‐drug induction regimen of prednisone, vincristine, daunorubicin, and asparaginase (PVDA). Following re‐induction, patients treated with regimen‐A underwent consolidation with a combination of teniposide and AraC, whereas those on regimen‐B received consolidation therapy with HD‐AraC and asparaginase. After consolidation, maintenance therapy was administered for 120 weeks, employing pairs of drugs to which the patients had not shown resistance. This maintenance regimen was pursued for patients not designated for HSCT. CNS‐directed radiation therapy was planned for all patients. Those with CNS involvement at relapse were administered craniospinal radiation, receiving 2400 cGy to the brain and 1200 cGy to the spine. Patients without CNS disease at the time of relapse were subjected to cranial radiation alone, with a dose of 1800 cGy.

#### 2.4.4 Statistical analysis

The analysis of data was conducted using IBM SPSS Statistics for Windows, version 20.0 (IBM Corp., Armonk, NY, USA). To ensure the integrity of the data, quality assurance protocols were implemented, focusing on the completeness and accuracy of the data collected. Summary statistics were generated and analyzed to establish baseline and demographic characteristics. These characteristics were delineated through the use of frequencies and percentages for categorical variables, and relevant variables were subjected to cross‐tabulation for further examination. For the measurement of follow‐up durations, statistical metrics, such as the mean, median, and range were employed, with all durations expressed in years. The study also conducted survival analysis using the Kaplan–Meier method. This approach facilitated the estimation of the 3‐year OS rates for the cohort, with the survival time calculated in years from the date of diagnosis to the last date seen or the date of death for deceased patients. To assess the significance of differences in survival outcomes, statistical tests—either the log‐rank or the Tarone–Ware test—were applied as appropriate. A *p*‐value of less than .05 was predetermined as the threshold for statistical significance.

## 3 RESULTS

### 3.1 Cohort analysis

The study cohort comprised 31 children, resulting in a relapse incidence of 9% (31 out of 347 patients) among children who either relapsed or were resistant to first‐line therapy and received salvage therapy. Out of the total study cohort (31), there are 25 male patients (83.33%), and 6 female patients (16.67%). The average age at diagnosis is 6.54 ± 3.4 years (median: 6 years, ranging from 1.5 to 13 years). Table 1 presents the clinical characteristics of the study cohort at the time of diagnosis. The majority of patients (84%, *n* = 26) exhibited a Pre‐B immunophenotype, while a smaller proportion (16%, *n* = 5) had a Pre‐T immunophenotype. Regarding white blood count (WBC) counts at diagnosis, 68% (*n* = 21) of the patients had counts below 50 000/μL, indicating a lower disease burden, whereas 32% (*n* = 10) presented with counts of 50 000/μL or above, suggesting a higher disease burden. The CNS status, a critical factor in assessing disease severity and treatment strategy, showed that most patients (87%, *n* = 27) were classified as CNS 1, indicating no detectable CNS involvement. A single patient (3%) was classified as CNS 2, and a small group (10%, *n* = 3) fell into the CNS 3 category, which denotes CNS involvement. Extramedullary disease was predominantly absent in the cohort, with 90% (*n* = 28) of patients showing no signs. However, a small number of patients exhibited extramedullary involvement, including testicular (7%, *n* = 2) and mediastinal (3%, *n* = 1) disease. Risk stratification revealed a majority of the patients (71%, *n* = 22) were classified as HR, while 29% (*n* = 9) were considered LR.

**TABLE 1:** Clinical characteristics of the cohort at diagnosis.

Clinical characteristics	At diagnosis % (*N*)
Immunophenotype	
Pre‐B	84% (26)
Pre‐T	16% (5)
WBC counts/μL	
<50 000	68% (21)
≥50 000	32% (10)
CNS status	
CNS 1	87% (27)
CNS 2	3% (1)
CNS 3	10% (3)
Extra medullary disease	
No	90% (28)
Testis	7% (2)
Mediastinal	3% (1)
Risk stratification	
LR	29% (9)
HR	71% (22)

Table 2 provides an overview of the clinical characteristics at relapse for the study cohort. Within the study cohort, three patients encountered IFs, while 28 experienced relapses. Of the relapses, 14% (4 out of 28) occurred during first‐line therapy, and the remainder, 86% (24 out of 28), happened after the completion of therapy. For those who relapsed, the duration of CR1 varied widely, from 2.3 to 99 months, with an average of 26 months and a median of 2 months. Specifically, 48% (15 patients) relapsed within 18 months, 23% (7 patients) between 18 and 36 months, and 29% (9 patients) after more than 36 months of achieving either IF or CR1. Regarding the site of relapse, 61% (19 patients) had isolated BM involvement, 23% (7 patients) experienced isolated extramedullary relapses—including 5 with CNS relapses and 2 with testicular relapses. The remaining 16% (5 patients) had BM involvement alongside other sites, with 4 cases of BM + CNS and one case of BM + testis involvement. At the point of relapse, risk stratification revealed that 6% (2 patients) were classified as LR, 23% (7 patients) as IR, and the majority, 71% (22 patients), were considered HR before receiving salvage therapy. Subsequently, 55% (17 patients) underwent first‐salvage chemotherapy, 45% (14 patients) received second‐salvage therapy, and 42% (13 patients) proceeded to undergo HSCT.

**TABLE 2:** Clinical characteristics of study cohort at relapse.

Clinical characteristics	At relapse % (N)
Remission status at relapse	
CR1	90% (28)
IF	10% (3)
Duration of IF or CR1 at relapse	
≤18 months	48% (15)
18–36 months	23% (7)
>36 months	29% (9)
Site of relapse	
BM	62% (19)
BM + other sites:	16% (5)
BM + CNS	13% (4)
BM + Testis	3% (1)
Isolated extra‐medullary:	23% (7)
CNS	16% (5)
Testis	6% (2)
Risk stratification at relapse	
Low risk (LR)	6% (2)
Intermediate risk (IR)	23% (7)
High risk (HR)	71% (22)
Salvage therapy received	
First‐salvage chemotherapy	55% (17)
Second‐salvage therapy	45% (14)
Hematopoietic stem cell transplantation (HSCT)	42% (13)

In the study, five patients received the UKALR3 regimen, which included dexamethasone, vincristine, mitoxantrone, peg‐asparaginase, and intrathecal methotrexate. Another five patients were treated with the FLAG regimen, comprising fludarabine, high‐dose AraC (cytarabine), and granulocyte colony‐stimulating factor (G‐CSF), with or without idarubicin. The REZ‐BFM (Relapse Berlin‐Frankfurt‐Münster) regimen, consisting of dexamethasone, mercaptopurine, methotrexate, AraC, and asparaginase, was administered to three patients. Similarly, three patients underwent treatment following the St. Jude Total XIII B HR protocol, while two patients received other salvage protocols—one with ifosfamide, carboplatin, etoposide (ICE) and another with the COG AALL0232 protocol. One patient opted out of therapy.

Patients classified as HR at relapse, as well as those categorized as IR or LR but resistant to the first line of salvage therapy, were considered eligible for allogeneic HSCT, contingent on the availability of a suitable donor. Among the 13 patients who underwent HSCT, ten were classified as HR, and three as IR at the time of relapse. The donor source for all these patients was HLA‐identical siblings. The conditioning regimen for the transplantation included cyclophosphamide (CY) and total body irradiation (TBI). Prophylaxis for graft‐versus‐host disease (GVHD) comprised methotrexate (MTX) and cyclosporine (CSA).

#### 3.1.1 Survival and remission status

**FIGURE 1:**
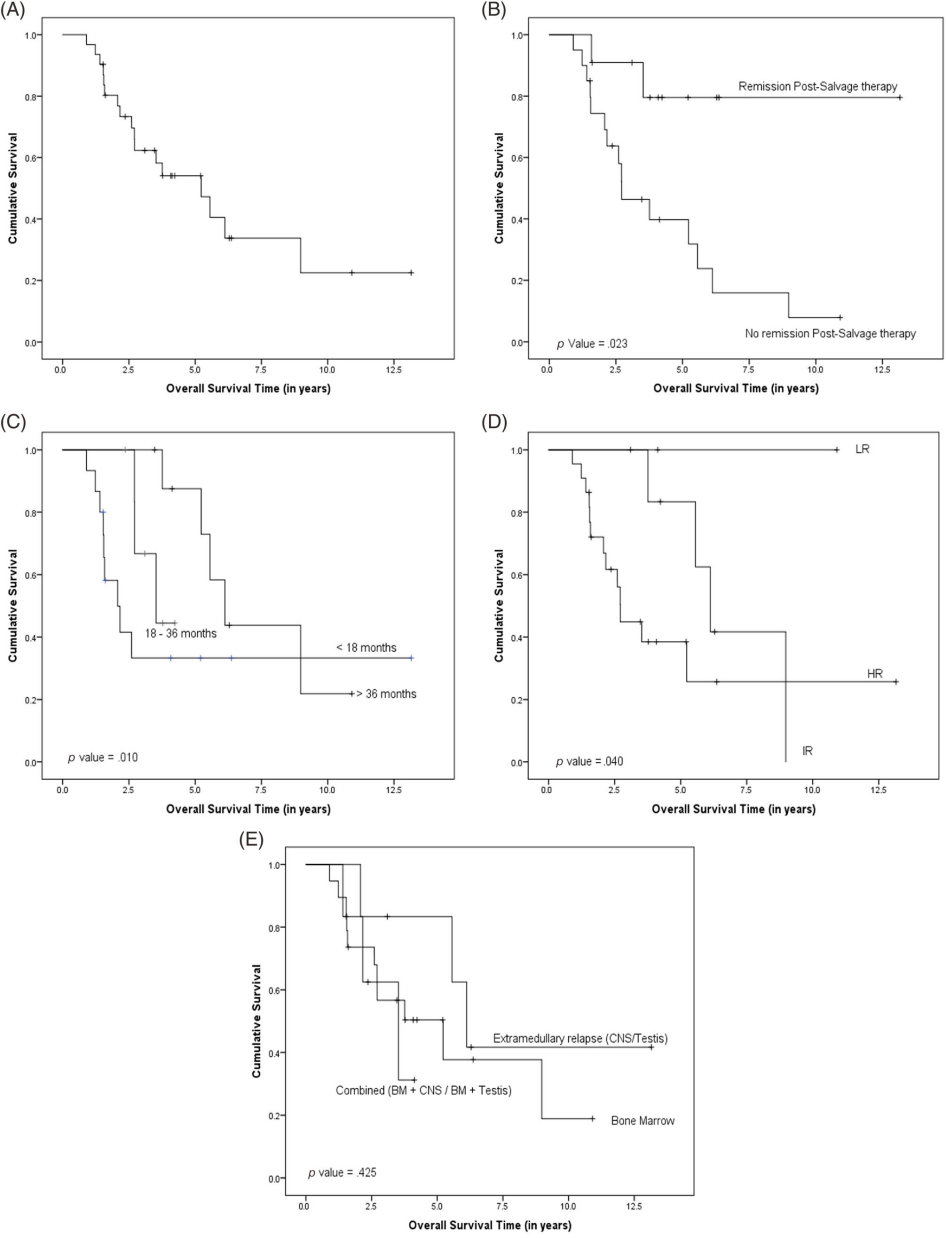
Overall survival of study cohort (*N* = 31), retrospective study: (A) Overall survival (OS) of the cohort; (B) Overall survival of cohort comparing patients who achieved CR2 with Salvage therapy versus those who did not achieve CR; (C) Overall survival of study cohort based on duration of CR1; (D) Overall survival of study cohort based on risk‐stratification at relapse; and (E) Overall survival of study cohort based on relapse site.

Within all relapse categories, 36% of patients (*n* = 11) achieved CR after the first salvage therapy, while 64% (*n* = 20) proceeded to second‐salvage therapy, achieving remission in 11 of 20 cases. The overall remission rate observed in the cohort was 71%. The 3‐year OS rate for all 31 patients was 62.3%, with a median follow‐up of 3.4 years (range: 0.9–13.2 years). Among those who attained CR after the first‐salvage therapy (*n* = 11), a 3‐year OS rate of 79.5% was observed, significantly higher compared with those who did not achieve CR after the first‐salvage therapy (3‐year OS: 46.4%) (*p*‐value <.05). Similarly, the duration of CR prior to relapse showed significant differences in survival rates: ≤18 months at 33.2%, 18–36 months at 66.7%, and >36 months at 87.5%, with these differences being statistically significant (*p*‐value <.05). This trend of statistical significance was also observed when comparing the 3‐year OS based on risk stratification at relapse: LR at 83.3%, IR at 80%, and HR at 44.8% (*p*‐value <.05). However, the 3‐year OS comparison based on the site of relapse showed no statistical significance: extramedullary relapse (CNS/testis) at 3.3%, BM at 56.7%, and combined sites (BM + CNS/BM + testis) at 62.5%. Survival outcomes are summarized in Figure 1.

### 3.2 Chemotherapy versus HSCT

**FIGURE 2:**
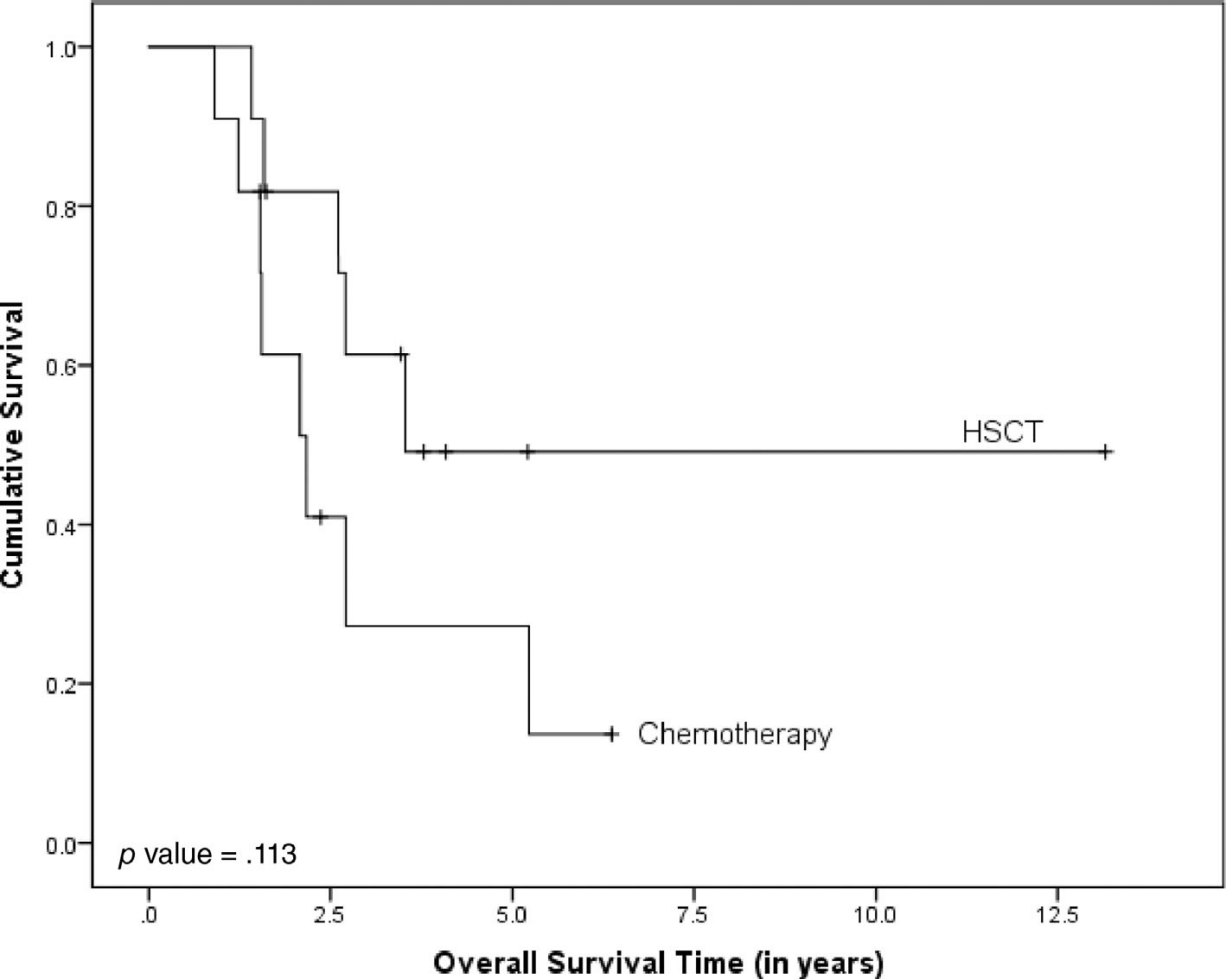
Overall survival of HR patients at relapse (*N* = 13) comparing patients who received HSCT versus those who received chemotherapy.

Thirteen patients underwent HSCT from fully matched, HLA‐identical siblings, while the rest of the cohort received chemotherapy as their sole treatment. All patients who received HSCT achieved complete donor‐derived engraftment. Despite this success, four patients experienced relapse, and three succumbed to transplant‐related mortality (TRM) while in remission. Among the 18 patients treated with chemotherapy alone, four relapsed, and 11 passed away due to treatment‐related toxicities. A comparison of the 3‐year OS rates between those who underwent HSCT and those who received chemotherapy (61.4% vs. 27.3%, respectively) is presented in Figure 2. However, the sample sizes for both the HSCT and chemotherapy groups are too small to conduct a statistically significant comparison.

## 4 DISCUSSION

Recent advancements in chemotherapy regimens have markedly improved the 5‐year survival rates for children newly diagnosed with ALL, with current survival rates reported at approximately 90% and cure rates ranging between 80% and 90% following initial treatment. Despite these achievements in frontline therapy, a subset of pediatric patients, accounting for 10%–15%, still face relapse and subsequently poor prognoses.^8^, ^9^, ^10^ The site and timing of relapse are consistently identified as critical determinants of survival following a relapse. In our study, we employ and illustrate a risk‐stratification approach to the treatment of children with relapsed or refractory ALL to report our risk‐factors and outcomes for survival post—relapse in our cohort of patients.

The relapse rate of 9% in our cohort is comparable to some reports while lower than that reported by various research groups and significantly lower than the relapse rates of 24.5% and 20.5% reported by Ali et al.^11^ and Nguyen et al.,^12^ respectively.^4^ The male‐to‐female ratio in our cohort was 4.16:1, mirroring the findings of several other studies that also noted a higher relapse rate among males compared with females, indicating a poor prognosis factor.^4^ Consistent with other studies, we showed a higher rate of relapse in the HR group of patients than LR patients (71% vs. 29%). In terms of relapse site, BM relapse was the most common, accounting for 61% of cases, a finding that is in agreement with several previous studies.^4^, ^13^, ^14^

Among the relapsed cases in our study, 64% of the patients (15 patients) did not achieve remission, a rate that is higher than what is typically reported in the literature.^4^, ^12^ Nevertheless, our study also shows early response to re‐induction therapy is of prognostic value. The remission failure in these patients may be attributed to the site of relapse—60% experienced isolated BM relapse, 25% had BM combined with CNS relapse, 10% had isolated CNS relapse, and one patient (5%) experienced testicular relapse. Our analysis suggests that the leukemia blast cells were likely inherently resistant to the agents used in first‐line therapy. Additionally, the lack of MRD testing at that time might have contributed to a sub‐optimal response to treatment, resulting in poorer outcomes. These observations align with findings from the second Therapeutic Advances in Childhood Leukemia Consortium (TACL) study, which reported a 51% CR rate among children with BM relapse.^6^

In our study, the correlation between OS and events of relapse revealed that the response to re‐induction therapy emerged as the predictor of survival within our cohort. Utilizing a risk‐stratification strategy that considered the duration of remission after first‐line therapy and the site of relapse, we identified a specific cohort with relatively good risk upon OS analysis after disease recurrence. This approach and its findings bear resemblance to those described by Belgaumi et al.,^3^ (particularly in identifying a cohort with a notably favorable prognosis characterized by an OS rate of 91.7% for patients with isolated extramedullary relapse beyond 18 months of CR1) and BM relapse beyond 12 months off‐therapy. Given limited number of patients who received allogeneic HSCT, patients only receiving matched sibling grafts, and lack of measures of minimal residual disease, we are not able to make any conclusion as to the benefit of HSCT in our cohort.

As of the cutoff for analysis in January 2021, 45% of the children who relapsed have deceased, while 55% remain alive, with a median survival time post‐relapse of 7.5 months. This survival duration aligns with the findings reported by Tuong et al.^4^ But is shorter than those noted in other studies.^15^, ^16^ This discrepancy could potentially be attributed to the absence of MRD testing to assess the response to second‐line therapy and the utilization or potential enhancement of treatment protocols and therapeutic agents.^6^

Several cellular immunotherapeutic modalities have demonstrated their efficacy in children with relapsed or chemotherapy‐refractory disease over the years.^17^ Blinatumomab is one of the first‐in‐class bispecific antibody therapeutics that has established its superiority compared with standard chemotherapy in patients with ALL, with a tolerable toxicity profile.^18^, ^19^ A multi‐institutional, open‐label, expanded access study conducted at 16 centers in Europe and the United States recommends the use of blinatumomab as a safe and efficacious treatment option in children with relapse and refractory ALL.^19^ Another multi‐canter, open‐label, randomized controlled phase 3 clinical trial demonstrates blinatumomab's efficacy in patients randomized to receive a third consolidation course with either one cycle of blinatumomab or three cycles of HR consolidation therapy, irrespective of the MRD status before treatment initiation. The study findings suggest the inducing role of blinatumomab in achieving MRD remission, negating the MRD prognostic value.^20^

Similarly, inotuzumab ozogamicin (InO) is another agent that is recognized as effective in relapsed ALL adult patients, and data on safety and efficacy in children are still emerging. One such retrospective study conducted in pediatric patients with relapsed or refractory ALL from a compassionate use program reports InO as being well‐tolerated and an effective therapeutic option in children with relapsed ALL.^21^ These findings were comparable to those experienced in the adult population, with a complete response (CR) rate of 67%. However, caution is needed for patient's proceeding to HSCT after InO due to risk of sinusoidal obstruction syndrome (SOS).^21^ A single‐arm, open‐label, phase II clinical trial of InO in children and adolescents with relapsed and refractory ALL by the COG reports InO as an effective and well‐tolerated agent in patients who have been heavily treated for ALL, with a CR rate of 58.3%. This study also noted SOS after HSCT.^22^ Therefore, blinatumomab and InO are now actively being investigated by the COG in multi‐center clinical trials for newly diagnosed ALL patients to be incorporated into standard chemotherapy regimens upfront based on risk stratification defined by clinical, biology‐based, and disease response.^23^

The study is not without limitations, as our analysis lacks data on socio‐demographic and physiological factors, organ function, co‐morbidities, and performance scoring. However, similar to the TACL study, our study provides background information for future outcome analysis in this patient population at our institution.^6^ Blinatumomab with MRD testing is a significant treatment advancement for children with relapsed or refractory leukemia,^24^ and this is another limitation of this data as the immunotherapeutic agents were not introduced at our institution until 2019. Therefore, a follow‐up multi‐center study to generate pooled data and compare CR rate and survival outcomes between the two‐time frames is underway, that is, after introducing novel therapeutic agents, such as blinatumomab, InO, and more recently CD19 CAR‐T cell therapy.

## 5 CONCLUSION

Our study shows the majority of relapse events occurred during the first 18 months from first remission, and BM remains the leading site of relapse. Risk stratification appears to be effective in identifying the “better risk” category, but response to re‐induction remained the strongest predictor for survival. Additional data with a larger sample size may help define optimal role and outcomes for HSCT in relapsed and refractory ALL. Therefore, a multi‐institutional observational study is recommended for a larger cohort of patients as a follow‐up to this study to compare survival outcomes and transplant‐related toxicity (infectious and noninfectious) in these groups of patients. Data from our cohort confirms the need for novel and alternative approaches to improve outcomes for relapsed and refractory ALL.

## AUTHOR CONTRIBUTIONS

*Study conception and design*: Mosfer AlMalki and Mohammed Abdulatef. *Data collection*: Mohammed Abdulatef and Hassan Altrabolsi. *Analysis and interpretation of results*: Mosfer AlMalki, Hassan Altrabolsi, and Nasser Shubayr. *Draft manuscript preparation*: Mosfer AlMalki and Nasser Shubayr. All authors reviewed the results and approved the final version of the manuscript.

## CONFLICT OF INTEREST STATEMENT

The authors have stated explicitly that there are no conflicts of interest in connection with this article.

## ETHICS STATEMENT

All procedures performed in this study involving human participants were in accordance with the ethical standards of the institutional and/or national research committee and the 1964 Declaration of Helsinki and its later amendments (or comparable ethical standards). This study was approved by the institutional review board of King Faisal Specialist Hospital and Research Centre, Jeddah, Saudi Arabia (IRB No.2021‐14).

## Data Availability

The data that support the findings of this study are available from the corresponding author upon reasonable request.
